# Strong CH…O Interactions in the Second Coordination Sphere of 1,10-Phenanthroline Complexes with Water

**DOI:** 10.3390/ijms262412100

**Published:** 2025-12-16

**Authors:** Sonja S. Zrilić, Jelena M. Živković, Dragan B. Ninković, Snežana D. Zarić

**Affiliations:** 1 Innovative Centre of the Faculty of Chemistry, Studentski trg 12-16, 11000 Belgrade, Serbia; sonjaz@chem.bg.ac.rs (S.S.Z.); andric_jelena@chem.bg.ac.rs (J.M.Ž.); 2 Institute of Chemistry, Technology and Metallurgy, University of Belgrade, Njegoševa 12, 11000 Belgrade, Serbia; dragan.ninkovic@ihtm.bg.ac.rs; 3 University of Belgrade—Faculty of Chemistry, Studentski trg 12-16, 11000 Belgrade, Serbia

**Keywords:** CH…O hydrogen bonds, 1,10-phenanthroline, transition metal complexes, Cambridge structural database, quantum chemical calculations

## Abstract

Although CH…O hydrogen bonds are generally very weak, here investigated CH…O interactions of coordinated 1,10-phenanthroline (phen) are very frequent and quite strong. In the crystal structures from the Cambridge Structural Database, 8344 CH…O interactions between coordinated phen and water molecule in the second coordination sphere were found. We calculated all possible types of CH…O interaction energies at DLPNO-CCSDT/CBS level for non-coordinated and coordinated phen with a water molecule. The data for non-coordinated phen exhibited the weakest interactions, from −2.09 to −2.94 kcal/mol. Upon coordination of phen, interactions become stronger. In octahedral cobalt(II) complexes, interaction energies are from −3.37 to −4.35 kcal/mol. With the decrease in the complex coordination number, interaction energies become stronger, the strongest are for square planar palladium(II) complexes from −3.91 to −4.94 kcal/mol. There is a linear correlation between interaction energies and electrostatic potential values at the interacting hydrogen atom, with a correlation coefficient of 0.97. For all studied systems, the weakest is always a linear interaction, and the strongest is a bifurcated interaction. The strongest calculated CH…O interactions of coordinated phen with water in the second coordination sphere (−4.94 kcal/mol) are as strong as the hydrogen bond between two water molecules (−5.0 kcal/mol).

## 1. Introduction

The CH…O interactions are considered to be very weak interactions, since the partial positive charge on the hydrogen atom is quite small due to the small difference in electronegativity between carbon and hydrogen [1,2,3,4]. The CH…O interactions are stronger in organic molecules containing heteroatoms [5,6]. For example, pyridine forms moderately strong interactions; the energy of the bifurcated pyridine/water interaction is −2.30 kcal/mol [6]. Also, the strength of CH…O interactions can be increased by substituents on the aromatic ring [7,8,9,10]. By choosing strong acceptors, one can obtain relatively strong CH…O interactions. In the 1,2,4,5-tetrafluorobenzene–acetone complex, an interaction energy of −3.2 kcal/mol [9] is obtained, while the energy of bifurcated interaction in the caffeine–theophylline complex is −4.64 kcal/mol [10].

It was observed that noncovalent interactions of aromatic molecules can strengthen the CH…O interactions. When a pyridine molecule involves a nitrogen atom in a classical hydrogen bond, it is capable of forming stronger CH…O interactions. As was mentioned above, the interaction energy of the bifurcated pyridine/water interaction is −2.30 kcal/mol [6]. However, water/pyridine/water trimer, with the first water/pyridine interaction being OH…N and the second pyridine/water interaction being CH…O, the second bifurcated CH…O interaction has the interaction energy of −2.69 kcal/mol [6].

The coordination of molecules influences classical hydrogen bonds [11,12,13,14,15,16]. For example, calculations of hydrogen bond energy showed that coordinated ethylenediamine forms a significantly stronger hydrogen bond [14]. Namely, when non-coordinated ethylenediamine forms hydrogen bonds with a water molecule, the interaction energy is −2.3 kcal/mol, while coordinated ethylenediamine in neutral complexes forms hydrogen bonds with the interaction energy of −4.0 to −6.7 kcal/mol, depending on the metal ion. The interactions are quite strong for positively charged complexes, from −8.5 kcal/mol for a singly positive complex and up to −28.0 kcal/mol for a triply positively charged complex.

**Figure 1:**
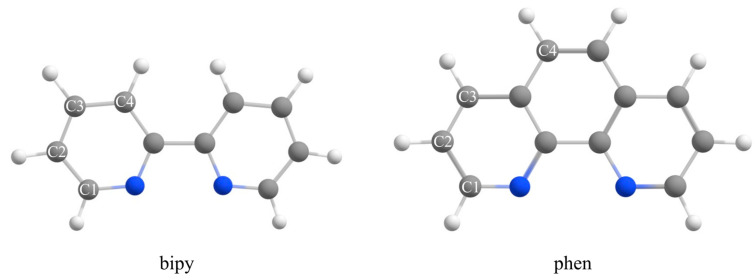
Structures of 2,2′-bipyridine (bipy) and 1,10-phenanthroline (phen) with marked C1, C2, C3, and C4 carbon atoms, which correspond to different types of CH groups. The standard color scheme is applied: hydrogen—white, carbon—grey, nitogen—blue.

Considering these data on the influence of coordination on classical hydrogen bonds, the question was whether coordination can influence CH…O hydrogen bonds. A recent study shows that coordination of aromatic molecules strengthens CH…O hydrogen bonds [17]. The study on the interaction energies and analysis of the data in the Cambridge Structural Database (CSD) showed that the strength of CH…O interactions of the 2,2′-bipyridine (bipy) molecule is remarkably stronger when bipy is coordinated. Bipy is an aromatic molecule that coordinates to a metal ion via nitrogen atoms (Figure 1). The calculations at a very accurate DLPNO-CCSD(T)/CBS level were performed on CH…O interactions of bipy complexes, as C-H donors, with water molecules, as C-H acceptors (Figure 1). The neutral complexes with coordination numbers six (cobalt complex), five (copper complex), and four (palladium complex) were used for the calculations. The C3-H…O interaction energy for non-coordinated bipy is −2.07 kcal/mol, while for coordinated bipy the energies are −3.49, −3.75, and −4.02 kcal/mol, for cobalt, copper, and palladium complexes, respectively. A bipy molecule can form bifurcated interactions, which are stronger than linear ones. The strongest bifurcated interaction, C2-C3 interaction, for non-coordinated bipy has an interaction energy of −2.20 kcal/mol. The strongest bifurcated interactions for coordinated bipy are C4-C4, with energies of −5.10, −5.41, and −5.80 kcal/mol for cobalt, copper, and palladium complexes, respectively. These data show that relatively weak CH…O interactions of non-coordinated bipy become quite strong by coordination. The data also show that the strength of CH…O interactions increases with decreasing coordination number; hence, the strongest interactions are of palladium complexes with coordination number four. The data from the CSD crystal structures support the results of the calculations. The analysis of the crystal structure data shows that CH…O interaction distances shorten with decreasing coordination number, while the angles indicate a preference for bifurcated interactions.

Similar to the aromatic bipy molecule, the 1,10-phenanthroline (phen) molecule can be coordinated to a metal ion by nitrogen atoms (Figure 1) and, similar to bipy, it is used as a common ligand in many metal complexes. The difference between bipy and phen ligands is that phen has extended aromaticity, and it is more rigid [18]. Because of it, metal complexes of phen are more stable, and some other properties of metal complexes are influenced, such as luminescence and catalytic activity [18]. Complexes with coordinated phen have been synthetized with a large number of metals [18,19]. These complexes show interesting supramolecular assemblies [20,21,22,23,24,25], biological activity [26,27,28,29], catalytic activity [30,31], and photophysical properties [32], and have been used in electrochemical reactions [33,34] and for chemosensing cations and anions [35]. It was shown that CH…O interactions of phen ligands stabilize supramolecular structures in crystals of dimeric copper and trimeric cobalt complexes [36], nickel complex [37], and cobalt complex [38].

Considering the importance of MOFs, the Nobel Prize in Chemistry for 2025 was given for the discovery of MOFs. A number of MOFs have been made using phen as a ligand, and these MOFs can be used as chemosensors because of their luminescence properties. Zn-MOF with phen ligand can be used as a chemosensor for metal ions (Fe(III) and Cu(II)), trinitrophenol, and colchicine [39]. Eu-MOFs can be used as chemosensors for Fe(III), Al (III), 2-hydroxy-1-naphthaldehyde [40], and colchicine [41]. A new class of Zn-MOFs can be used to determine glucose in human samples [42]. Eu-Ru-MOF has the capability to catalyze the reduction of CO_2_ by visible-light-driven reduction [43].

One can hypothesize that CH…O interactions can stabilize many supramolecular structures and can be important in catalysis to bind substrates with electronegative atoms, as was recognized in the crystal structure of a cobalt complex where sulfur oxoanions were forming CH…O interactions with phen ligand [38]. The aim of our study was to show the existence of CH…O interactions in crystal structures of phen complexes, to evaluate the strength of these interactions, and the factors that influence the strength. In accordance with this aim, we studied CH…O interactions of non-coordinated and coordinated phen molecule for different positions of hydrogen atoms (Figure 1) by analyzing all crystal structures in the CSD and by performing quantum chemical calculations on model systems. The CH…O interactions were studied for complexes with different coordination numbers. To the best of our knowledge, for the first time, CH…O interactions of non-coordinated and coordinated phen molecules were studied.

## 2. Results

### 2.1. CSD Search Results

**Figure 2:**
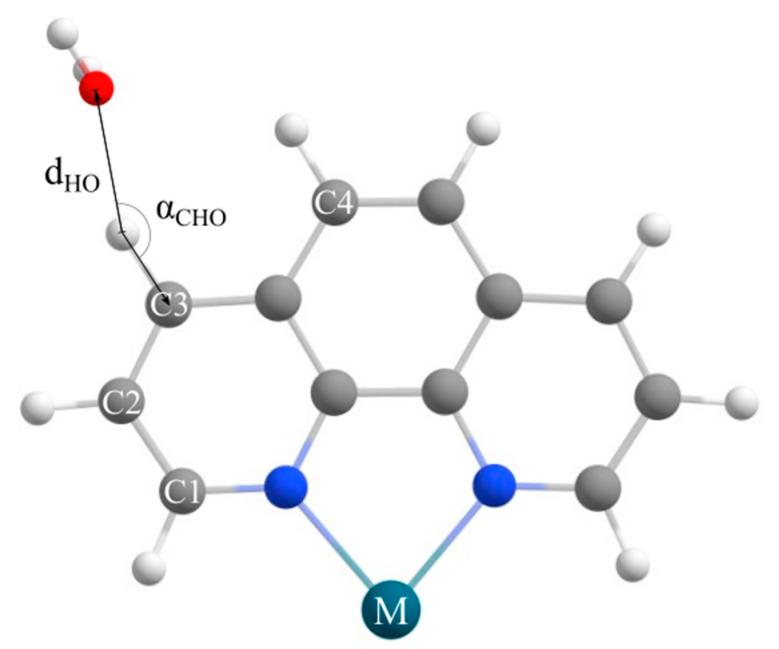
The geometrical parameters used for the CSD search. The d_HO_ (Å) denotes the distance between hydrogen and oxygen atoms, while α_CHO_ (˚) angle denotes CHO angle. The notation C1-C4 on coordinated phenantroline (phen) denotes the positions of the carbon atoms that can be involved in CH…O interactions. The M symbol denotes a metal ion.

Analysis of crystal structures revealed a large number of CH…O interactions in systems containing phen complexes and free (non-coordinated) water molecules, with 8344 interactions meeting the geometrical criteria. The CSD search was performed for CH…O hydrogen bonds for three different types of CH…O hydrogen bonds via C2, C3, and C4 (Figure 2). The C1 position was not considered, since other ligands in the complex are close to C1 and there is a possibility for bifurcated interactions involving other ligands (Figure 2).

**Figure 3:**
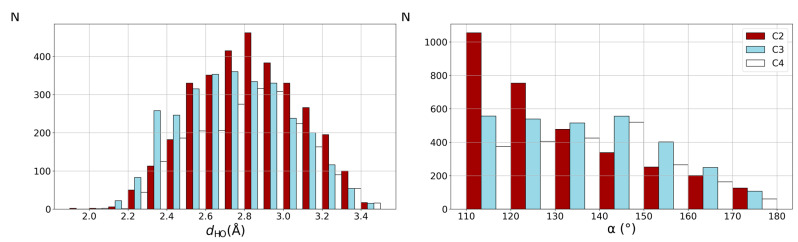
Distribution of angle α (**left**) and d_HO_ distances (Å) (**right**) for CH…O interactions of phen complexes and water.

Distributions of hydrogen bond distance (d_HO_) and angle (α) are presented in Figure 3. Additional results separated depending on the phen complex geometry to coordination number 6 (octahedral), 5 (trigonal bipyramidal or square pyramidal), and 4 (square planar or tetrahedral) are presented in the Supplementary Information (Figure S2).

The most commonly observed geometry among the analyzed complexes is octahedral, corresponding to a coordination number 6, with a total of 5159 interactions (Figure S1). A relatively large number of structures correspond to coordination number 5, adopting either trigonal bipyramidal or square pyramidal geometries (2212 interactions). Additionally, a considerable number of square planar structures with coordination number 4 were identified (245 interactions), and a nearly identical occurrence was found for structures with coordination number 9, 263 interactions (Figure S1). While the number of interactions for coordination number 4 and coordination number 9 is comparable, the square planar geometry associated with coordination number 4 is of higher interest due to its prevalence in coordination chemistry. Consequently, further investigation by DFT calculations will focus on coordination numbers 6, 5, and 4. 

Among the coordination number 6, the majority are cobalt complexes (1111 interactions). Within the coordination number 5, copper complexes are the most dominant (2037 interactions), while for coordination number 4, palladium complexes are most dominant (127 interactions) (Figures S3–S5).

### 2.2. QM Calculations

**Figure 4:**
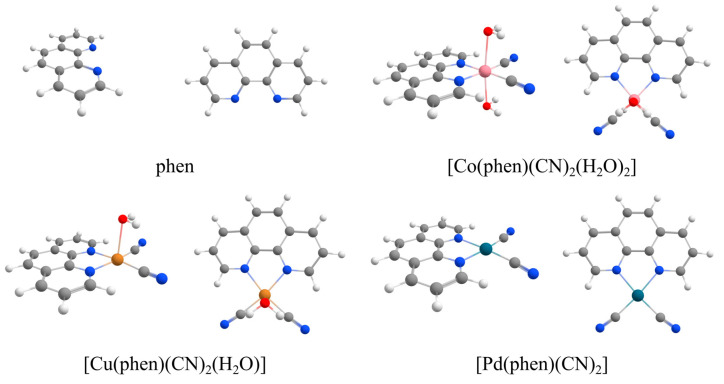
Side and top views of phen and cobalt(II), copper(II), and palladium(II) phen complexes after geometry optimization. The coordinates are given in the Supplementary Information.

**Figure 5:**
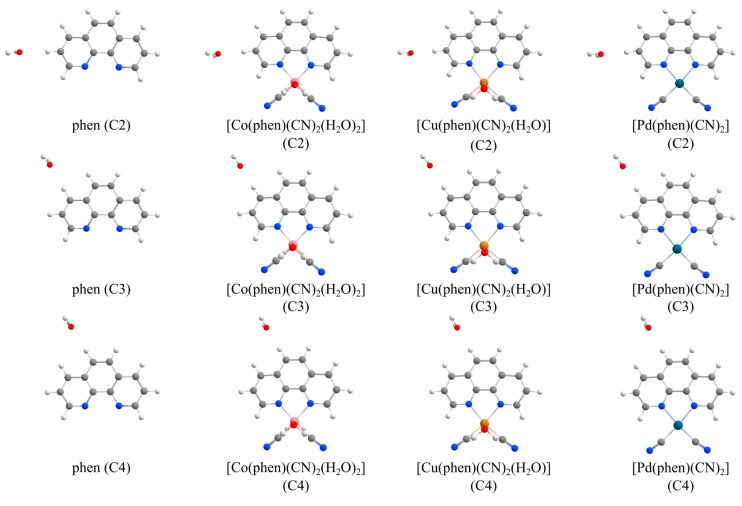
The linear CH…O hydrogen bonds for phen and its cobalt(II), copper(II), and palladium(II) complexes. The coordinates are given in the Supplementary Information.

**Figure 6:**
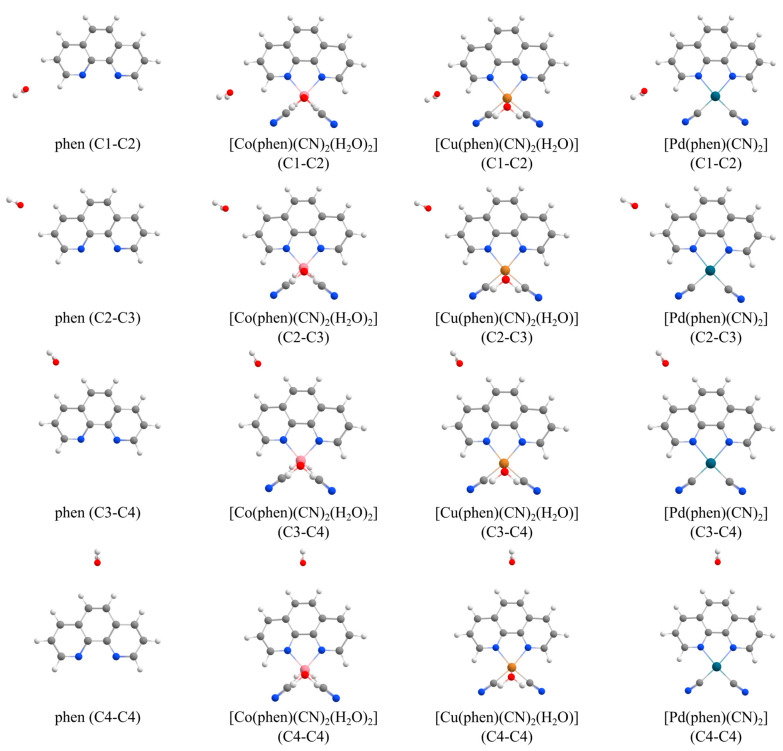
The bifurcated CH…O hydrogen bonds for phen and its cobalt(II), copper(II), and palladium(II) complexes. The coordinates are given in the Supplementary Information.

Phen and its complexes are optimized, and their geometries are shown in Figure 4. Different types of CH…O hydrogen bonds were calculated for each of them: linear hydrogen bonds via C2, C3, and C4 carbon atoms (Figure 5), as well as bifurcated interactions via C1-C2, C2-C3, C3-C4, and C4-C4 (Figure 6). Potential energy curves were calculated for all linear and bifurcated CH…O hydrogen bonds by performing a single-point calculation for various hydrogen bond distances with a 0.1 step (Figure S6). After performing the benchmark study comparing 19 methods to DLPNO-CCSDT/CBS level of theory (Tables S1 and S2), B3LYP-D4/def2-TZVP was chosen due to its very high accuracy and efficiency (Table S3). The distance and energy of the strongest interaction along the potential energy curve at B3LYP-D4/def2-TZVP and level are reported for each CH…O interaction type (Table 1 and Table 2).

**Figure 7:**
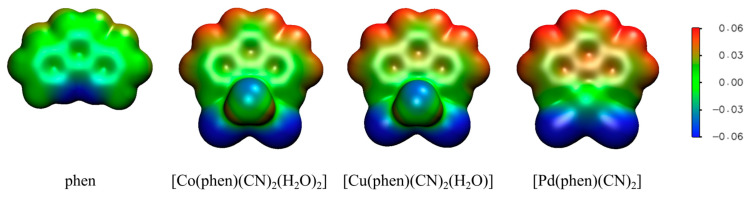
Electrostatic potential mapped on 0.001 au electron density surface for phen, its cobalt(II), copper(II), and palladium(II) complexes. Color scale shows that red regions have positive, green near 0, and blue negative electrostatic potential.

Electrostatic potentials on 0.001 au electron density surfaces at the B3LYP-D4/def2-TZVP level calculated for phen and its complexes are shown in Figure 7, while values of electrostatic potentials on interacting hydrogen atoms (V_S_) are shown in Table 1 and Table 2.

Lastly, geometry optimization and interaction energy of the optimized systems were calculated at the B3LYP-D4/def2-TZVP level starting from the geometries that are minima on potential curves obtained by single-point calculations. The results for optimized systems are presented in Table S4 and Figure S7.

**Figure 8:**
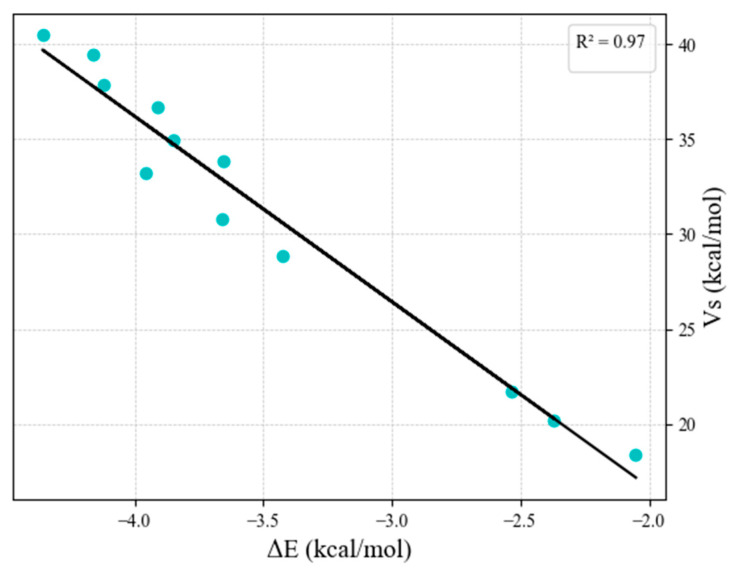
Linear dependence of interaction energy ΔE (kcal/mol) and electrostatic potential values V_S_ (kcal/mol) on interacting hydrogen atoms, calculated at B3LYP-D4/def2-TZVP level for linear CH…O hydrogen bonds of non-coordinated and coordinated phen.

We also presented the dependence of interaction energy ΔE and electrostatic potential V_S_, both calculated at the B3LYP-D4/def2-TZVP level. Figure 8 shows the linear dependence of interaction energy ΔE and electrostatic potential V_S_ with a correlation coefficient of 0.97 for linear CH…O hydrogen bonds of both non-coordinated and all coordinated phen with water.

## 3. Discussion

### 3.1. CSD Discussion

Across all three coordination numbers (six, five and four), one can notice similar distributions of α angle and d_HO_ distance (Figure S1). Hence, distributions for each coordination number were presented in the Supplementray Information (Figure S2), while the distribution for all contacts is presented in Figure 3. The α angle distributions show a clear tendency toward small values. These results indicate that the majority of CH…O interactions are likely bifurcated, which can be attributed to the close spatial arrangement of hydrogen atoms in aromatic phen molecules. Among the three positions, C2 has a larger tendency towards quite small angles in the range of 110–130°, whereas C3 and C4 adopt wider ranges of α angle (Figure 3).

This behavior is consistent with previous findings on CH…O hydrogen bonding in aromatic systems such as benzene and pyridine [6,44], as well as for coordinated bipy [17], where steric proximity of adjacent hydrogens promotes bifurcated geometries of the CH…O interactions.

The distribution of d_HO_ distance for CH…O interactions shows a clear distribution of values in the range of 2.7–3.0 Å. These distances are somewhat longer than bifurcated CH…O hydrogen bonding in coordinated bipy ligand [17] and calculated data for phen complexes in Table 2. In coordinated bipy ligands, calculated d_HO_ values for bifurcated interactions are often between 2.4 and 2.6 Å [17], while calculated data for phen ligand are 2.5 to 2.6 Å. A somewhat longer distance can be explained by additional simultaneous interactions of water molecules in the crystal structures. Figure 3 also shows that distances shorter than 2.4 Å are relatively rare, while contacts beyond 3.2 Å gradually decrease in frequency, reflecting weaker and less geometrically favorable interactions.

### 3.2. QM Discussion 

Data in Table 1 show that for non-coordinated phen, linear hydrogen bond distances d_HO_ (Figure 5) are 2.3–2.4 Å and bifurcated CH…O interactions (Figure 6) for C1-C2 and C2-C3 distances d_HO_ are 2.7 Å, while C3-C4 bifurcated interaction is shorter, with 2.5 Å distance (Table 1). Interaction energies at DLPNO-CCSDT/CBS level are −2.09, −2.57, and −2.44 kcal/mol for linear CH…O interaction types, respectively. Bifurcated hydrogen bonds lead to stronger interactions. The strongest interaction energy of −2.94 kcal/mol is obtained for C3-C4 CH…O hydrogen bond type, while other bifurcated interaction energy values are −2.12, −2.34, and −2.32 kcal/mol (Table 1).

Benchmark study revealed that out of 19 levels, 5 are in excellent agreement with DLPNO-CCSDT/CBS single-point interaction energies with mean error 0.06 kcal/mol or lower and maximum error under 0.20 kcal/mol across all systems and interaction types (Tables S1–S3). Among these five methods, we chose B3LYP-D4/def2-TZVP because it is also the fastest among them (Table S3). Table 1 shows that interaction energies at the B3LYP-D4/def2-TZVP level closely follow DLPNO-CCSDT/CBS results and lead to the same conclusions.

Coordinated phen has stronger and mainly shorter interactions than non-coordinated ones (Table 1 and Table 2). For the cobalt(II) octahedral complex, interaction energies of linear interactions at DLPNO-CCSDT/CBS level are −3.37, −3.84, and −3.63 kcal/mol, and their d_HO_ distances are all 2.3 Å (Table 2). The strongest CH…O interaction energy for this complex and water is again calculated for a bifurcated C3-C4 type, and its value is −4.35 kcal/mol. This interaction type and C1-C2 have 2.5 Å long hydrogen bonds, while the other two have d_HO_ distances of 2.6 Å. The remaining bifurcated interaction energies are −3.57, −3.78, and −3.51 kcal/mol (Table 2).

For the copper(II) square pyramidal complex, all CH…O hydrogen bonds are stronger than the corresponding values for the cobalt(II) complex. Linear hydrogen bonds have DLPNO-CCSDT/CBS interaction energies −3.59, −4.08, and −3.86 kcal/mol, and 2.3 Å d_HO_ distances. In case of bifurcated CH…O hydrogen bonds, C3-C4 type shows the strongest hydrogen bond with interaction energy of −4.61 kcal/mol, and hydrogen bond length of 2.5 Å. Interaction energies and d_HO_ distances for other bifurcated interactions are −3.82 kcal/mol and 2.5 Å for C1-C2, −4.01 kcal/mol and 2.6 Å for C2-C3, and −3.74 kcal/mol and 2.6 Å for C4-C4 (Table 2).

The square planar palladium(II) complex has the strongest interaction energy for each type of CH…O hydrogen bond. Hydrogen bonds at DLPNO-CCSDT/CBS level for linear interactions are −3.91, −4.42, and −4.17 kcal/mol, and d_HO_ distances are 2.2 Å and 2.3 Å. The interaction energy of −4.94 kcal/mol, obtained for the bifurcated CH…O interaction of C3-C4 type of palladium(II) complex with water, is the strongest hydrogen bond among all results obtained for minima on potential curves calculated by single-point calculations (Table 2). The remaining bifurcated hydrogen bonds of palladium(II) complex are also strong, and their values are −4.37 kcal/mol, −4.33 kcal/mol, and −4.02 kcal/mol. Hydrogen bond distances d_HO_ are 2.5 Å and 2.6 Å (Table 2). For coordinated phen systems, B3LYP-D4/def2-TZVP interaction energy values are very accurate, and all trends are the same as for DLPNO-CCSDT/CBS method (Table 2).

One can notice that the changes in interaction energies are the consequence of the positive potential of the interacting hydrogen atoms. The dependence of interaction energy ΔE (kcal/mol) and electrostatic potential values V_S_ (kcal/mol) at the B3LYP-D4/def2-TZVP level show a linear regression with a very high correlation coefficient of 0.97 (Figure 8). Hence, we can explain the calculated difference in interaction energies by differences in the electrostatic potential. The interactions with hydrogen atoms on C3 are the strongest for linear, while for the bifurcated, the strongest interaction also includes hydrogen on C3. This is valid for non-coordinated phen and for all studied complexes. This is in accordance with the calculated electrostatic potential on interacting hydrogen atoms, V_S_; the hydrogen atom on C3 has the most positive potential (Table 1 and Table 2). The weakest interactions are interactions with hydrogen on C2, again as a consequence of the lowest value of electrostatic potential (Table 1 and Table 2).

Strengthening of CH…O hydrogen bonds with coordination of phen and with the decrease in metal coordination number are all consequences of larger positive electrostatic potential on all C-H groups, as one can observe in Figure 7, and in electrostatic potential values V_S_ those given in Table 1 and Table 2. Upon coordination, the positive charge of the metal ion is shared with ligands, resulting in more positive electrostatic potentials on phen. Moreover, the smaller the coordination number, the more positive potential is transferred to each ligand, resulting in the square planar complex having the most positive potentials on phen, followed by the square pyramidal, and, lastly, the octahedral complex.

After geometry optimization, the water molecule has bifurcated interactions except for non-coordinated phen and water, where C2 and C4 interactions at the B3LYP-D4/def2-TZVP level do not change significantly (Table S4, Figure S7). These interactions are only slightly stronger than the energies obtained for minima on potential curves obtained by single-point calculation presented in Table 1. The strongest interaction after geometry optimization is again the C3-C4 bifurcated interaction of square planar palladium(II) complex and water with interaction energy −5.00 kcal/mol. Starting the geometry optimization of coordinated phen and water from the C2 or C1-C2 positions resulted in bifurcated interactions between C1 and cyanido ligand, which are expectedly much stronger and give interaction energies up to −11.36 kcal/mol.

Non-coordinated phen displays stronger DLPNO-CCSDT/CBS CH…O interactions than non-coordinated bipy [17]. Compared to coordinated bipy, coordinated phen exhibits stronger interaction energies for all types of CH…O except linear C4 and bifurcated C4-C4, which are significantly stronger for bipy [17]. These differences are in agreement with the electrostatic potential values on these groups (Table 1 and Table S5); for the C2 position, V_S_ values are the same, for C3, they are stronger for phen, while for the C4 position, they are significantly stronger for bipy. Slightly stronger C2 interaction energies for phen are the consequence of more positive electrostatic potentials in the vicinity. Moreover, a much more favorable orientation of C-H groups for C4-C4 bifurcated interaction for bipy than phen (Figure 1) is an additional reason why bipy has much strong interactions of this type.

## 4. Methods 

### 4.1. CSD Search

A search of the crystal structures archived in the Cambridge Structural Database (CSD, version 5.46, November 2024 release) [45] was carried out using the ConQuest program (version 2024.3.0) [46]. The objective was to identify crystal structures of free and coordinated phen interacting with water molecules through CH…O interactions (Figure 2). Specifically, CH…O interactions involving carbon atoms at positions C2, C3, and C4 were examined (Figure 2). Interactions at the C1 position were not considered due to the proximity of the coordinating metal center, which significantly affects the geometry of the investigated interaction. The applied geometric criteria for these hydrogen bonds were a donor–acceptor distance (C···O) less than 4.0 Å and a bond angle (α) greater than 110° (Figure 3). In addition, only structures in which the free water molecule has an H–O–H angle between 96.4° and 112.8° were taken into account [47].

### 4.2. QM Methods

All calculations were performed using the ORCA 6.0.1 program [48,49]. Phen, its transition metal complexes and a water molecule were optimized using the TPSS-D3BJ/def2-TZVP method [50,51,52,53,54]. Vibrational analysis was performed for all obtained stationary points, and it was confirmed that they are all local energy minima. Chosen phen complexes were neutral octahedral cobalt(II) [Co(phen)(CN)_2_(H_2_O)_2_], square pyramidal copper(II) [Cu(phen)(CN)_2_(H_2_O)], and square planar palladium(II) complex [Pd(phen)(CN)_2_]. Cobalt(II) and copper(II) complexes were doublets, while the palladium(II) complex and non-coordinated phen were singlets. Both cobalt(II) and copper(II) doublets are shown to be at least 20 kcal/mol more stable than their quartet counterparts.

The following types of CH…O hydrogen bonds were investigated for phen and all three transition metal complexes with a water molecule: linear, as well as bifurcated interactions (Figure 5 and Figure 6). Using the TPSS-D3BJ/def2-TZVP method, interaction energy profiles were calculated with a 0.1 Å d_HO_ step using rigid monomers of phen or its complex and a water molecule in single-point calculations. The interaction energy profiles were calculated by changing the d_HO_ distance from 1.9 to 2.7 Å for C2, C3, and C4, and from 2.1 to 3.0 Å for bifurcated interactions. All interaction energies are corrected for basis set superposition error (BSSE) [55]:

$$∆E_{int}=E_{AB}-E_{A}^{AB}-E_{B}^{AB}$$

where $∆E_{int}$ is the total interaction energy, $E_{AB}$ is the energy of dimer *AB*, while $E_{A}^{AB}$ and $E_{B}^{AB}$ are energies of monomers *A* and *B*, calculated using the basis set for both *A* and *B* fragments.

For the geometry that corresponds to the minima on the interaction energy curve obtained by single-point calculations, the benchmark study was performed to find the best DFT functional for these interactions, and the results are shown in Tables S1–S3. In the benchmark study, 19 different methods were compared to the values calculated with the DLPNO-CCSDT method with a complete basis set (CBS), using the extrapolation method with def2-SVP and def2-TZVP basis sets [56,57,58,59,60,61,62,63,64]. Table S3 shows that the B3LYP functional with D4 dispersion correction is one of the most accurate tested methods, and, additionally, very fast. Interaction energy profiles were recalculated at the B3LYP-D4/def2-TZVP level (Figure S6) and at the DLPNO-CCSDT/CBS level for those systems where interaction energy minima were changed. The results of interaction energy minima at both DLPNO-CCSDT/CBS and B3LYP-D4/def2-TZVP levels, as well as hydrogen bond distances d_HO_ for single-point calculations with rigid monomers, are reported in Table 1 for non-coordinated phen with water, and in Table 2 for coordinated phen with water.

Electrostatic potentials at the B3LYP-D4/def2-TZVP level on 0.001 au of electron density surfaces, as proposed by Bader et al. [65], were calculated for phen and its complexes using the ORCA 6.0.1 program [48,49] and visualized with VMD 1.9.3. software [66]. The values of electrostatic potentials on interacting hydrogen atoms (V_S_) in the direction of the hydrogen bond were calculated for each linear CH…O type of each system (Table 1 and Table 2). In order to be able to compare these values to the data from our previous work on CH…O hydrogen bonds of bipy [17], V_S_ values are calculated in the same manner for non-coordinated and coordinated bipy (Table S5).

Starting from the geometry of the minima on potential curves obtained by single-point calculations of each system, geometry optimization at the B3LYP-D4/def2-TZVP level, followed by vibrational analysis, was performed to find the preferred position of the water molecule with respect to free or coordinated phen. Interaction energies at the same level of theory were calculated between phen and water in optimized systems. Hydrogen bond distances and interaction energies for optimized systems are shown in Table S4 and geometries of these systems in Figure S7.

## 5. Conclusions

Although CH…O hydrogen bonds are generally very weak, all investigated CH…O interactions of the coordinated 1,10-phenanthroline (phen) molecule are quite strong. In this work, we studied the interaction of non-coordinated and coordinated phen molecules with water molecules by searching crystal structures in the CSD and by performing DLPNO-CCSDT/CBS and B3LYP-D4/def2-TZVP calculations.

The data from CSD crystal structures show a large number of CH…O interactions between coordinated phen and water molecule in the second coordination sphere, 8344 contacts with hydrogen atoms on C2, C3, and C4. The distribution of the α angle indicates dominantly bifurcated interactions.

Calculations were performed for three linear hydrogen bonds and four bifurcated hydrogen bonds with a water molecule in the second coordination sphere. Coordination of the phen molecule to a metal ion causes CH…O interactions to be significantly stronger than for a non-coordinated phen molecule. Non-coordinated phen has interaction energies at DLPNO-CCSDT/CBS level in the range from −2.09 to −2.94 kcal/mol, while coordinated phen are in the range from −3.37 to −4.94 kcal/mol.

Our results show that with decreasing the complex coordination number, interaction energies become stronger. For octahedral cobalt(II) complexes, interaction energies are from −3.37 to −4.35 kcal/mol, for square pyramidal copper(II) complexes, they are from −3.59 to −4.61 kcal/mol, and for square planar palladium(II) complexes, from −3.91 to −4.94 kcal/mol. The decrease in coordination number leads to a larger fraction of the metal ion’s positive charge being shared with the phen ligand, which results in an increase in the positive electrostatic potential values on interacting hydrogens and stronger CH…O hydrogen bonds.

This trend for the influence of coordination number on hydrogen bonds closely follows the trend of electrostatic potentials on interactions between hydrogen atoms, which is also indicated by a linear correlation between interaction energies and electrostatic potential values with a correlation coefficient of 0.97.

The strongest calculated CH…O hydrogen bond for coordinated phen with the water in the second coordination sphere (−4.94 kcal/mol) is as strong as the hydrogen bond between two water molecules (−5.00 kcal/mol) [67], indicating that coordinated phen forms quite strong CH…O hydrogen bonds.

Our results can be important in understanding of properties of phen complexes, especially their contact with the surrounding, as in cases of supramolecular structures and where phen complexes show biological and catalytic activity. The patterns that our data show can be used to predict properties of phen complexes, including phen fragments in MOFs.

## Figures and Tables

**Table 1:** Hydrogen bond distances d_HO_ (Å), interaction energies ΔE (kcal/mol) at DLPNO-CCSDT/CBS (ΔE^CC^) and B3LYP-D4/def2-TZVP level (ΔE^B3LYP^), and electrostatic potentials V_S_ (kcal/mol) at B3LYP-D4/def2-TZVP level for CH…O hydrogen bonds between non-coordinated phen and water of linear and bifurcated type. The corresponding geometries are presented in Figure 5 and Figure 6.

CH…O Type	Non-Coordinated Phen			
Interacting C	d_HO_	ΔE^CC^	ΔE^B3LYP^	V_S_
C2	2.4	−2.09	−2.05	18
C3	2.3	−2.57	−2.53	22
C4	2.4	−2.44	−2.37	20
C1-C2	2.7	−2.12	−2.09	/
C2-C3	2.7	−2.34	−2.28	/
C3-C4	2.5	−2.94	−2.94	/
C4-C4	2.7	−2.32	−2.13	/

**Table 2:** Hydrogen bond distances d_HO_ (Å), interaction energies ΔE (kcal/mol) at DLPNO-CCSDT/CBS (ΔE^CC^), and B3LYP-D4/def2-TZVP level (ΔE^B3LYP^), and electrostatic potentials V_S_ (kcal/mol) at B3LYP-D4/def2-TZVP level for CH…O hydrogen bonds between coordinated phen and water of linear and bifurcated type. The corresponding geometries are presented in Figure 5 and Figure 6.

CH…O Type	Coordinated Phen											
	Coordination Number 6 [Co(phen)(CN)_2_(H_2_O)_2_]				Coordination Number 5 [Cu(phen)(CN)_2_(H_2_O)]				Coordination Number 4 [Pd(phen)(CN)_2_]			
Interacting C	d_HO_	ΔE^CC^	ΔE^B3LYP^	V_S_	d_HO_	ΔE^CC^	ΔE^B3LYP^	V_S_	d_HO_	ΔE^CC^	ΔE^B3LYP^	V_S_
C2	2.3	−3.37	−3.42	29	2.3	−3.59	−3.66	31	2.2	−3.91	−3.96	33
C3	2.3	−3.84	−3.85	35	2.3	−4.08	−4.12	38	2.3	−4.42	−4.36	40
C4	2.3	−3.63	−3.65	34	2.3	−3.86	−3.91	37	2.3	−4.17	−4.16	39
C1-C2	2.5	−3.57	−3.53	/	2.5	−3.77	−3.82	/	2.5	−4.37	−4.30	/
C2-C3	2.6	−3.78	−3.72	/	2.6	−3.98	−4.01	/	2.6	−4.33	−4.26	/
C3-C4	2.5	−4.35	−4.41	/	2.5	−4.61	−4.70	/	2.5	−4.94	−4.96	/
C4-C4	2.6	−3.51	−3.48	/	2.6	−3.74	−3.75	/	2.6	−4.02	−4.04	/

## Data Availability

The original contributions presented in this study are included in the article/Supplementary Materials. Further inquiries can be directed to the corresponding author.

## Supplementary Materials

The following supporting information can be downloaded at: https://www.mdpi.com/article/10.3390/ijms262412100/s1.

## Abbreviations

The following abbreviations are used in this manuscript:

phen	1,10-phenanthroline
bipy	2,2′-bipyridine
DFT	Density Functional Theory
CCSDT	Coupled Cluster with Single, Double, and Triple Excitations
CBS	Complete Basis Set
DLPNO	Domain-based Local Pair Natural Orbital
