# Supplementary material for: Strong CH…O Interactions in the Second Coordination Sphere of 1,10-Phenanthroline Complexes with Water
Source: Int J Mol Sci. 2025 Dec 16;26(24):12100. doi: 10.3390/ijms262412100 (PMC12732878; doi:10.3390/ijms262412100)
Supplement: Supplementary file 1 [file ijms-26-12100-s001.zip › ijms-3985944-supplementary.pdf]

Supplementary to

Strong CH…O Interactions in the Second Coordination Sphere of 1,10-Phenanthroline Complexes with Water

Sonja S. Zrilić[1], Jelena M. Živković[1], Dragan B. Ninković[2*], Snežana D. Zarić[3*]

1. CSD search

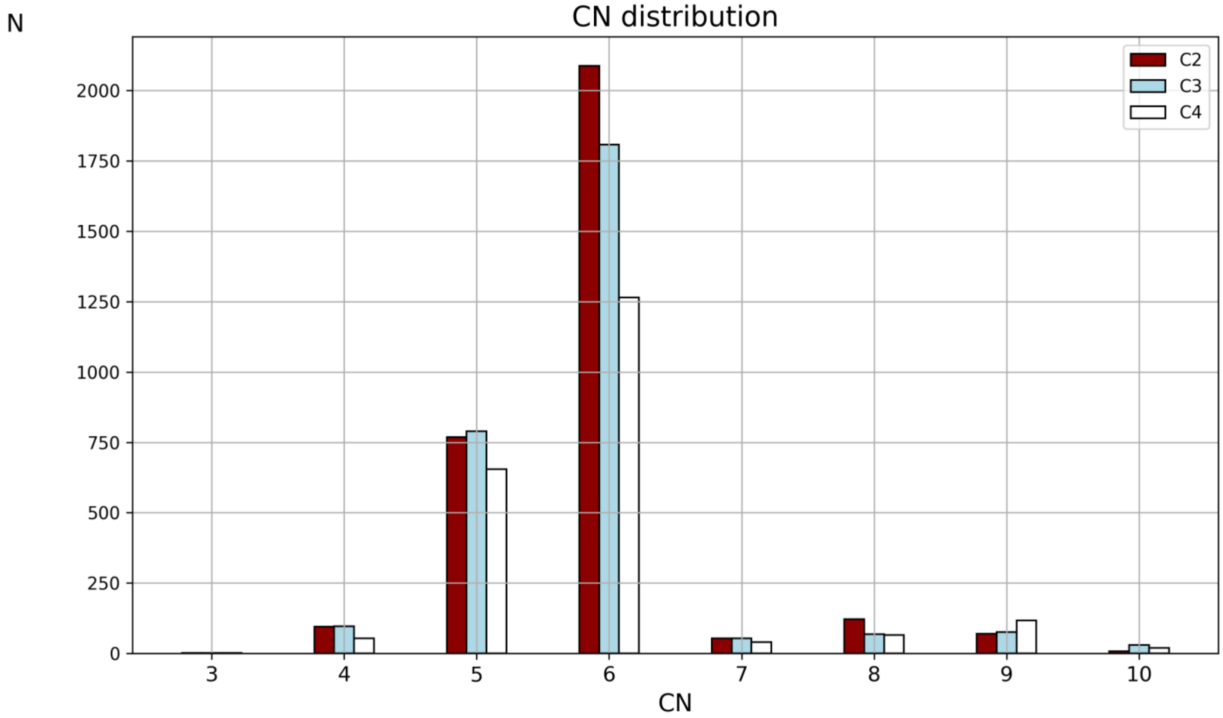

Figure S1. The distribution of coordination numbers (CN) for coordinated phen and water molecule.

For coordination number 6 complexes, the α angle distribution reveals maximum for C2 and C3 positions within the 110–120° range. Beyond this region, the number of interactions gradually declines; however, C4 shows not very pronaunced maximum at larger angles, between 140–150° (Figure S2). The distribution of α angle peaks for coordination number 5 complexes is in broader range than that observed for coordination number 6. The peak for C2 position again occurs within the 110–120° interval. However, C3 and C4 positions have maximam in the 140–150° range (Figure S2). For coordination number 4 complexes, the α angle distribution exhibits a maximum at 110–120° for the C2 position. The C3 and C4 positions display distinct patterns: C3 shows a maximum in the 140–150° range, while C4 is characterized by a broader maximum extending from 130 to 150° (Figure S2).

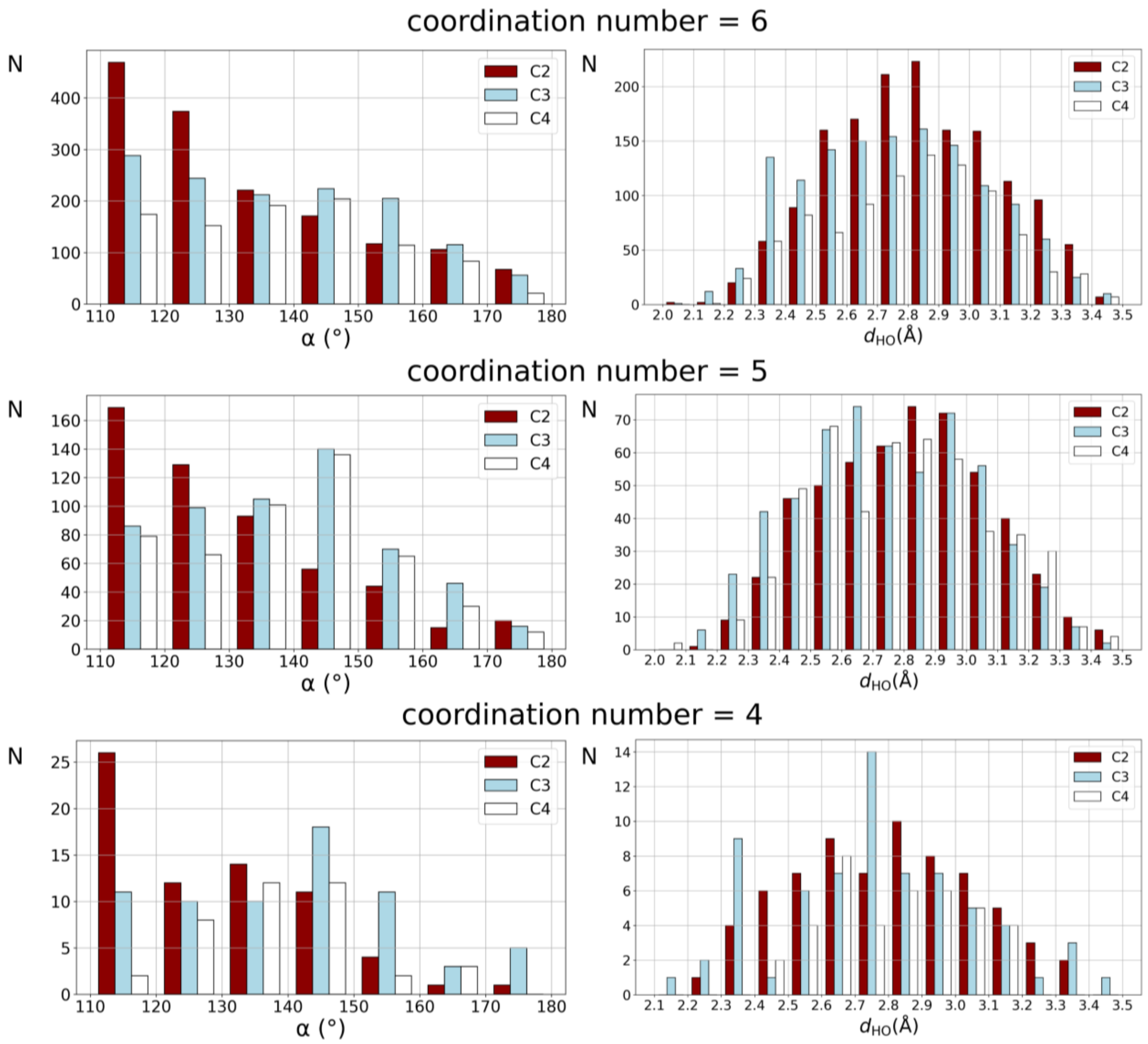

Figure S2. Distribution of angle α (left) and d_{HO} distances (Å) (right) for CH…O interactions of coordinated phen ligands in complexes with coordination numbers six, five, and four.

For coordination number 6 complexes most of the contacts for C2 have distance between 2.5–3.1 Å, with the peak in the range from 2.7 Å to 2.9 Å. Most of the contacts for C3 have tendency for shorter distances, in the range 2.3 to 3.0 Å, without pronounced peak, while most of the contacts for C4 are in the range 2.4 to 3.1 Å, with two peaks, one smaller at 2.4 to 2.5 Å and the other larger at 2.8 to 2.9 Å (Figure S2).

For coordination number 5 complexes the highest frequency occurs between 2.5–3.0 Å, with all three positions (C2, C3, and C4). The peak for C2 is at the range of 2.8-3.0 Å, the two peaks for C3 are 2.5 to 2.7 Å and C4 is at the range of 2.5 to 2.6 Å and 2.7-2.9 Å (Figure S2).

Unlike coordination number 5 and coordination number 6, for coordination number 4 complexes, the d_{HO} distribution shows somewhat different distribution, due to the smallest number of contacts. The most of contacts for C2 have distance in the 2.4 to 3.1 Å, with not very pronounced peak at 2.8–2.9 Å, contacts with C3 have two peaks, one at quite short distance 2.3-2.4 Å, and larger peak at 2.7-2.8 Å. Contacts with C4 have two peaks 2.6-2.7 Å and 2.8-3.0 Å (Figure S2).

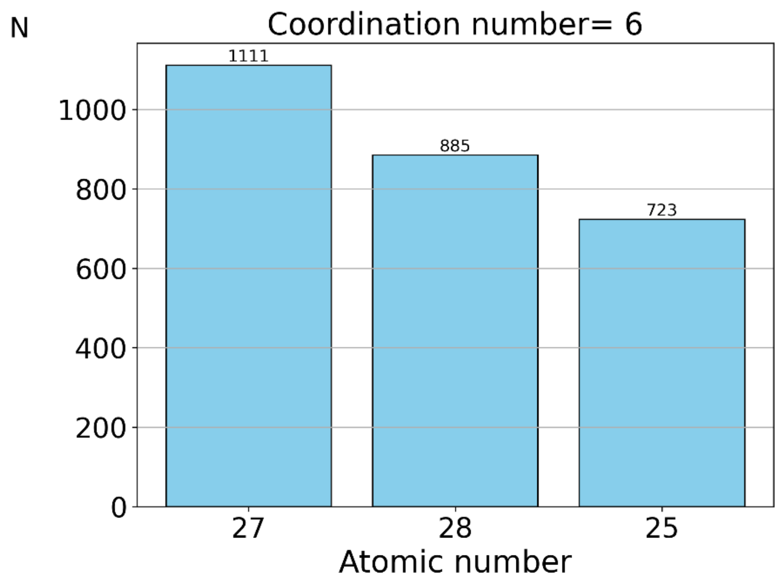

Figure S3. The three most abundant metals in the CSD for coordination number 6.

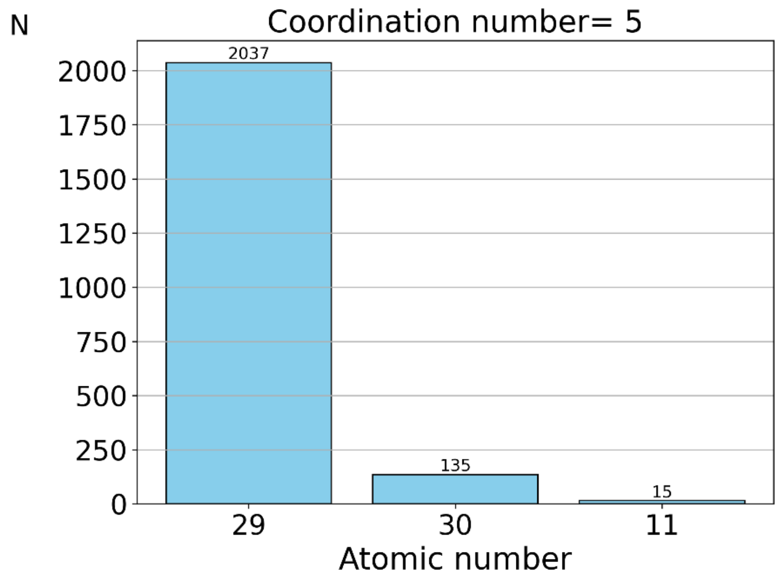

Figure S4. The three most abundant metals in the CSD for coordination number 5.

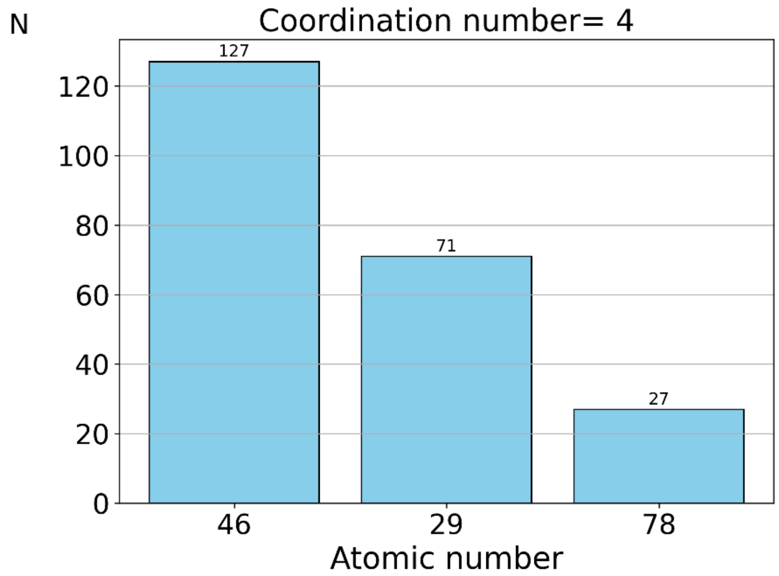

Figure S5. The three most abundant metals in the CSD for coordination number 4.

2. Benchmark study

Table S1. The benchmark study. Interaction energies (kcal/mol) at DLPNO-CCSDT/CBS level and various DFT methods with def2-TZVP basis set for non-coordinated phen and cobalt(II) phen complexes.

	**Non-coordinated phen**							**Coordinated phen Coordination number 6 [Co(phen)(CN)$_2$(H$_2$O)$_2$]**						
CH…O type	C2	C3	C4	C1C2	C2C3	C3C4	C4C4	C2	C3	C4	C1C2	C2C3	C3C4	C4C4
DLPNO-CCSDT	-2.086	-2.581	-2.442	-2.102	-2.304	-2.954	-2.279	-3.368	-3.840	-3.626	-3.554	-3.795	-4.352	-3.513
TPSS-D3BJ	-2.028	-2.498	-2.353	-1.966	-2.150	-2.801	-2.037	-3.179	-3.521	-3.413	-2.881	-3.281	-3.976	-3.146
TPSS-D4	-1.995	-2.46	-2.315	-1.967	-2.143	-2.778	-2.025	-3.191	-3.557	-3.429	-3.193	-3.378	-4.037	-3.219
TPSS-NL	-2.191	-2.674	-2.533	-2.142	-2.332	-3.025	-2.221	-3.370	-3.757	-3.635	-3.441	-3.566	-4.287	-3.420
M06L-D4	-1.433	-2.024	-1.883	-1.700	-1.892	-2.396	-1.782	-2.585	-3.076	-2.945	-3.030	-3.077	-3.645	-2.920
B3LYP-D4	-2.052	-2.521	-2.370	-2.032	-2.222	-2.895	-2.090	-3.425	-3.851	-3.653	-3.515	-3.689	-4.411	-3.462
B3LYP-NL	-2.249	-2.737	-2.589	-2.196	-2.401	-3.134	-2.280	-3.593	-4.041	-3.850	-3.728	-3.853	-4.635	-3.644
PBE0-D4	-2.301	-2.809	-2.655	-2.259	-2.439	-3.202	-2.301	-3.657	-4.131	-3.923	-3.729	-3.890	-4.704	-3.669
PBE0-NL	-2.392	-2.903	-2.751	-2.343	-2.531	-3.315	-2.399	-3.735	-4.213	-4.010	-3.848	-3.983	-4.817	-3.768
M062X-D3	-2.094	-2.632	-2.476	-2.243	-	-3.061	-2.264	-3.432	-3.968	-3.737	-3.893	-3.948	-4.602	-3.690
TPSSh-D4	-2.036	-2.515	-2.364	-2.009	-2.182	-2.839	-2.053	-3.313	-3.725	-3.56	-3.337	-3.522	-4.211	-3.326
TPSSh-NL	-2.207	-2.698	-2.55	-2.163	-2.348	-3.054	-2.226	-3.469	-3.895	-3.736	-3.555	-3.689	-4.429	-3.505
CAM-B3LYP-D4	-2.363	-2.875	-2.711	-2.338	-2.528	-3.291	-2.373	-3.792	-4.294	-4.049	-3.965	-4.087	-4.902	-3.811
wB97X-D4	-2.515	-3.070	-2.901	-2.565	-2.738	-3.582	-2.561	-3.879	-4.440	-4.188	-4.213	-4.296	-5.196	-4.014
wB97X-NL	-2.241	-2.784	-2.612	-2.328	-2.516	-3.218	-2.353	-3.577	-4.120	-3.863	-3.888	-4.018	-4.737	-3.737
B2PLYP-D4	-2.073	-2.537	-2.385	-2.052	-2.231	-2.895	-2.100	-3.440	-3.860	-3.668	-3.503	-3.705	-4.396	-3.481
B2PLYP-NL	-2.185	-2.665	-2.516	-2.158	-2.350	-3.041	-2.229	-3.549	-3.979	-3.791	-3.668	-3.825	-4.543	-3.611
PWPB95-D4	-2.054	-2.530	-2.367	-2.126	-2.300	-2.934	-2.132	-3.351	-3.799	-3.592	-3.604	-3.794	-4.412	-3.560
PWPB95-NL	-2.047	-2.528	-2.364	-2.093	-2.275	-2.915	-2.112	-3.319	-3.769	-3.561	-3.555	-3.739	-4.359	-3.510
wB97X-3c	-2.061	-2.586	-2.419	-2.198	-2.378	-3.002	-2.224	-3.399	-3.941	-3.676	-3.681	-3.885	-4.514	-3.602

Table S2. The benchmark study. Interaction energies (kcal/mol) at DLPNO-CCSDT/CBS level and various DFT methods with def2-TZVP basis set for copper(II) and palladium(II) phen complexes.

	Coordinated phen													
	Coordination number 5 $[Cu(phen)(CN)_2(H_2O)]$							Coordination number 4 $[Pd(phen)(CN)_2]$						
CH…O type	C2	C3	C4	C1C2	C2C3	C3C4	C4C4	C2	C3	C4	C1C2	C2C3	C3C4	C4C4
DLPNO-CCSDT	-3.592	-4.079	-3.865	-3.766	-3.980	-4.612	-3.745	-3.914	-4.418	-4.167	-4.369	-4.332	-4.935	-4.019
TPSS-D3BJ	-3.507	-3.946	-3.777	-3.482	-3.713	-4.423	-3.511	-3.779	-4.168	-4.015	-3.928	-3.961	-4.672	-3.740
TPSS-D4	-3.523	-3.954	-3.785	-3.568	-3.752	-4.451	-3.542	-3.794	-4.169	-4.017	-4.009	-4.000	-4.694	-3.765
TPSS-NL	-3.697	-4.150	-3.988	-3.805	-3.936	-4.701	-3.740	-4.010	-4.372	-4.226	-4.312	-4.245	-4.949	-3.968
M06L-D4	-	-3.427	-3.258	-3.364	-3.391	-4.000	-3.193	-3.149	-3.629	-3.476	-3.822	-3.662	-4.224	-3.395
B3LYP-D4	-3.661	-4.122	-3.910	-3.784	-3.950	-4.695	-3.695	-3.958	-4.358	-4.159	-4.305	-4.262	-4.957	-3.931
B3LYP-NL	-3.825	-4.309	-4.105	-3.986	-4.112	-4.920	-3.875	-4.161	-4.552	-4.361	-4.565	-4.479	-5.188	-4.117
PBE0-D4	-3.879	-4.388	-4.174	-3.990	-4.142	-4.974	-3.889	-4.203	-4.631	-4.429	-4.525	-4.473	-5.244	-4.131
PBE0-NL	-3.954	-4.468	-4.258	-4.100	-4.231	-5.086	-3.986	-4.299	-4.714	-4.516	-4.671	-4.599	-5.358	-4.231
M062X-D3	-3.619	-	-3.944	-4.124	-4.150	-4.818	-3.876	-3.875	-4.386	-4.166	-4.718	-4.539	-5.052	-4.085
TPSSh-D4	-3.587	-4.047	-3.855	-3.652	-3.828	-4.548	-3.593	-3.869	-4.273	-4.096	-4.114	-4.093	-4.802	-3.824
TPSSh-NL	-3.739	-4.215	-4.029	-3.861	-3.993	-4.767	-3.771	-4.059	-4.446	-4.275	-4.386	-4.318	-5.025	-4.006
CAM-B3LYP-D4	-3.995	-4.522	-4.270	-4.208	-4.308	-5.141	-4.013	-4.356	-4.790	-4.547	-4.821	-4.704	-5.436	-4.273
wB97X-D4	-4.069	-4.658	-4.396	-4.463	-4.506	-5.418	-4.200	-4.451	-4.936	-4.680	-5.107	-4.959	-5.722	-4.466
wB97X-NL	-3.761	-4.328	-4.064	-4.110	-4.220	-4.957	-3.919	-4.062	-4.605	-4.347	-4.655	-4.585	-5.259	-4.185
B2PLYP-D4	-3.654	-4.104	-3.901	-3.746	-3.942	-4.653	-3.694	-3.941	-4.341	-4.151	-4.266	-4.249	-4.917	-3.931
B2PLYP-NL	-3.759	-4.221	-4.023	-3.901	-4.058	-4.799	-3.822	-4.062	-4.460	-4.275	-4.447	-4.394	-5.064	-4.061
PWPB95-D4	-3.559	-4.038	-3.817	-3.883	-4.027	-4.661	-3.764	-3.819	-4.276	-4.066	-4.349	-4.363	-4.928	-4.001
PWPB95-NL	-3.524	-4.005	-3.785	-3.822	-3.970	-4.608	-3.713	-3.781	-4.246	-4.036	-4.282	-4.299	-4.876	-3.952
wB97X-3c	-3.591	-4.150	-3.880	-3.928	-4.087	-4.735	-3.787	-3.848	-4.406	-4.144	-4.394	-4.394	-5.019	-4.036

Table S3. Results of the benchmark study. Mean interaction energy error (kcal/mol), maximum error (kcal/mol), and average time (min) for each method from Tables S1 and S2 that was successful for all systems.

Method	Mean error	Maximum error	Average time	Method	Mean error	Maximum error	Average time
TPSS-D3BJ	0.24	0.67	1.8	CAM-B3LYP-D4	0.36	0.55	3.8
TPSS-D4	0.21	0.42	1.9	wB97X-D4	0.56	0.84	4.5
TPSS-NL	0.07	0.23	2.0	wB97X-NL	0.23	0.39	6.3
B3LYP-D4	0.06	0.19	3.4	B2PLYP-D4	0.05	0.18	4.2
B3LYP-NL	0.17	0.31	3.4	B2PLYP-NL	0.11	0.19	4.3
PBE0-D4	0.22	0.36	3.0	PWPB95-D4	0.05	0.15	4.2
PBE0-NL	0.32	0.47	3.0	PWPB95-NL	0.06	0.17	4.3
TPSSh-D4	0.12	0.27	3.2	wB97X-3c	0.06	0.16	4.4
TPSSh-NL	0.08	0.16	3.3				

3. Interaction energy curves

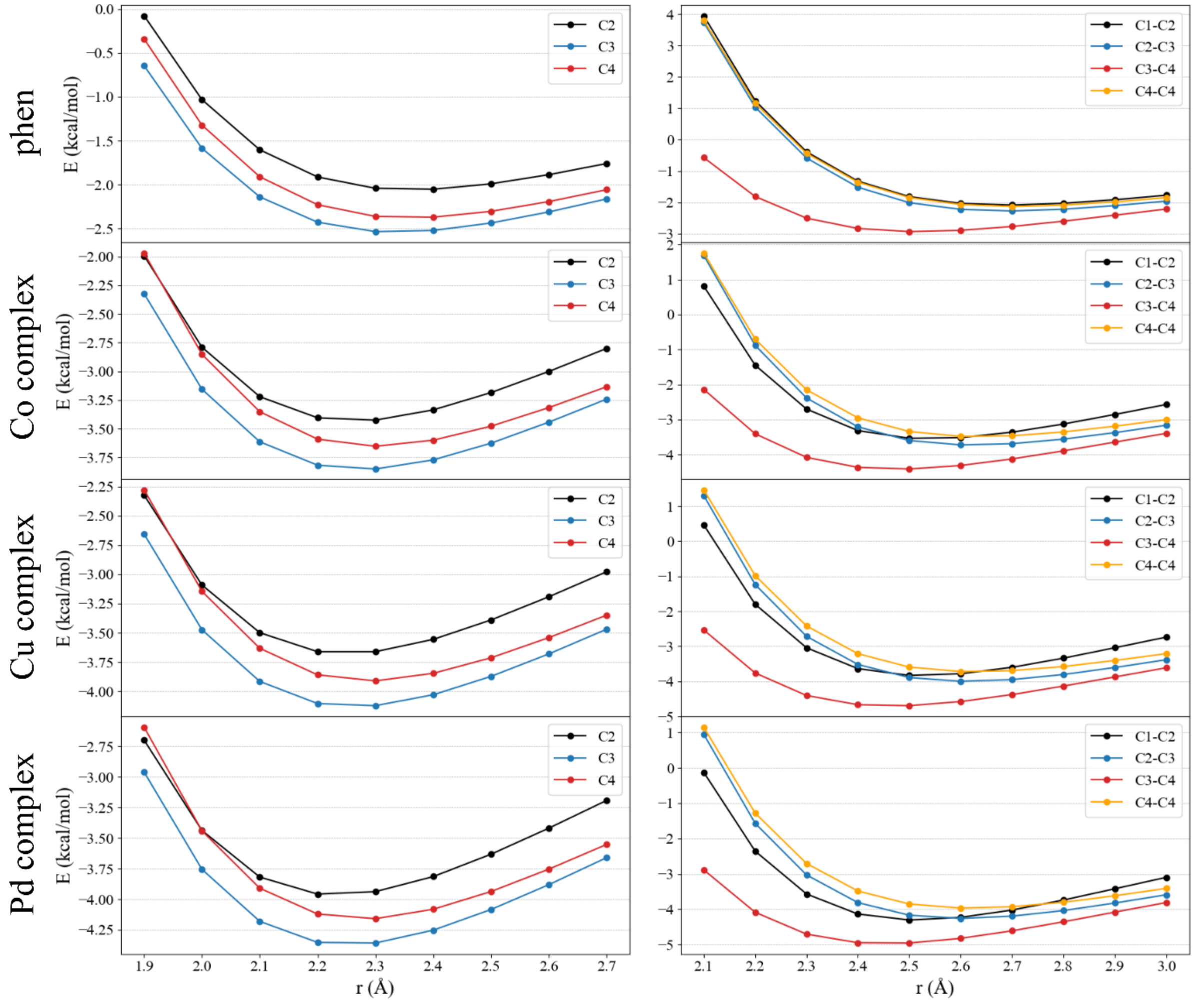

Figure S6. Interaction energy curves for non-coordinated and coordinated phen with water with the change of d_{HO} distance for linear (left) and bifurcated (right) CH…O interactions.

4. Total geometry optimization

Table S4. Geometry optimization results: interaction energies ΔE (kcal/mol) at B3LYP-D4/def2-TZVP level and types of interactions in the final geometry.

**CH…O type**	**Non-coordinated phen**		**Coordinated phen**					
			Coordination number 6 $[Co(phen)(CN)_2(H_2O)_2]$		Coordination number 5 $[Cu(phen)(CN)_2(H_2O)]$		Coordination number 4 $[Pd(phen)(CN)_2]$	
Interacting C	final geometry	ΔE	final geometry	ΔE	final geometry	ΔE	final geometry	ΔE
C2	C2	-2.08	CN-C1	-10.89	CN-C1	-10.77	CN-C1	-11.36
C3	C3-C4	-2.90	C3-C4	-4.44	C3-C4	-4.70	C3-C4	-4.99
C4	C4	-2.36	C3-C4	-4.44	C3-C4	-4.70	C3-C4	-4.99
C1-C2	C1-C2	-2.05	CN-C1	-10.89	CN-C1	-10.77	CN-C1	-11.35
C2-C3	C2-C3	-2.34	C2-C3	-3.85	C2-C3	-4.11	C2-C3	-4.39
C3-C4	C3-C4	-2.91	C3-C4	-4.45	C3-C4	-4.70	C3-C4	-5.00
C4-C4	C4-C4	-2.19	C4-C4	-3.56	C4-C4	-3.77	C4-C4	-4.04

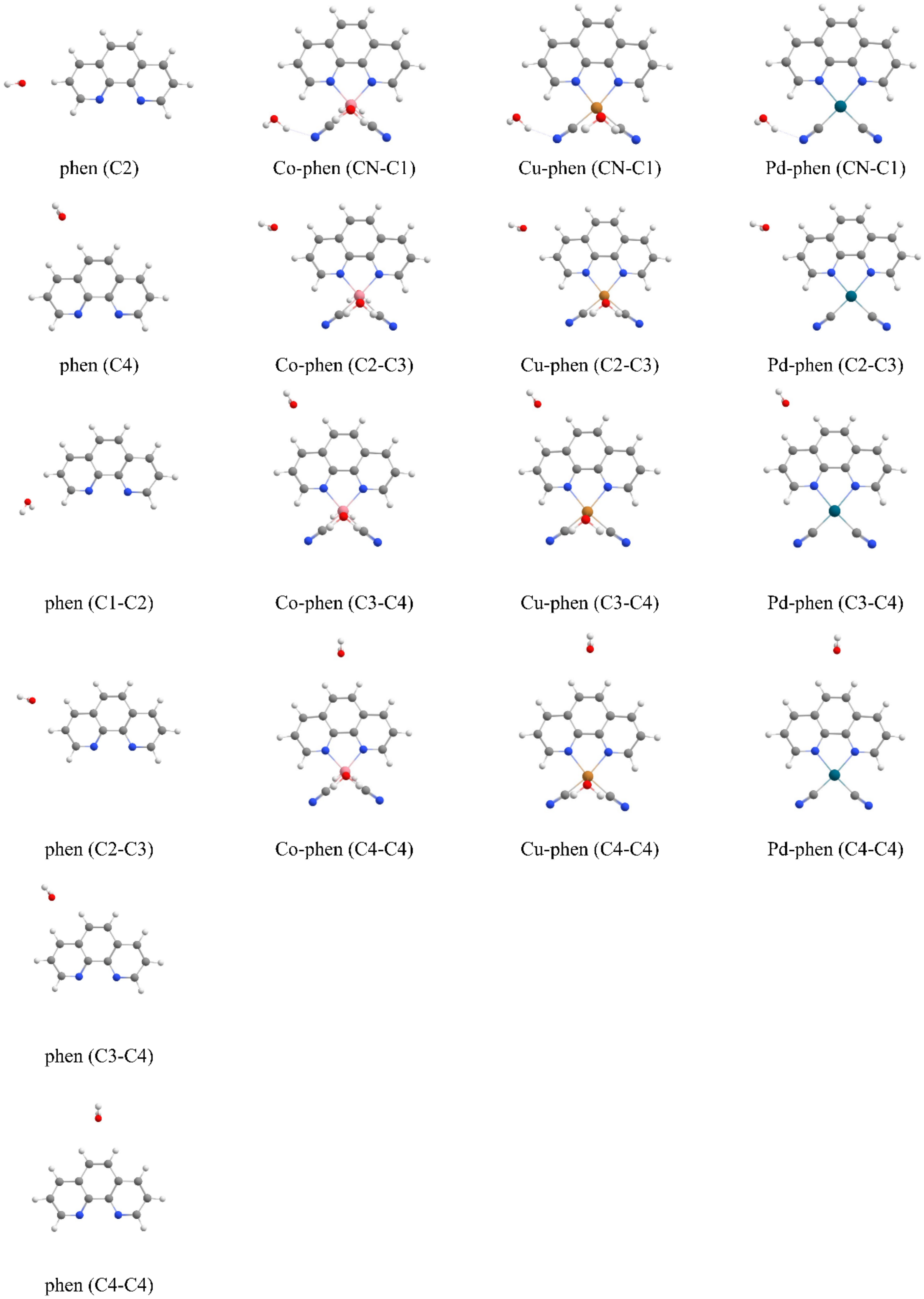

Figure S7. Types of interactions after geometry optimization of rigid systems.

5. Results for 2,2′-bipyridine

Table S5. Hydrogen bond distances d_{HO} (Å), and electrostatic potentials V_S (kcal/mol) at B3LYP-D4/def2-TZVP level for linear CH…O hydrogen bonds between coordinated 2,2′-bipyridine (bipy) and water of C2, C3, C4 type.

**CH…O type**	**Non-coordinated bipy**		**Coordinated bipy**					
			Coordination number 6 $[Co(bipy)(CN)_2(H_2O)_2]$		Coordination number 5 $[Cu(bipy)(CN)_2(H_2O)]$		Coordination number 4 $[Pd(bipy)(CN)_2]$	
Interacting C	d_{HO}	V_S	d_{HO}	V_S	d_{HO}	V_S	d_{HO}	V_S
C2	2.4	18	2.3	29	2.3	31	2.2	33
C3	2.4	18	2.3	34	2.3	37	2.3	39
C4	/	/	2.4	42	2.4	45	2.4	49

6. Coordinates of rigid systems

phen

C	1.355360	-3.018020	-0.119800
C	2.423910	-2.100020	-0.227740
C	2.147490	-0.755840	-0.299550
C	0.802600	-0.319220	-0.264010
C	-0.221800	-1.280970	-0.155440
N	0.083230	-2.623280	-0.085130
C	-1.603640	-0.840060	-0.118620
C	-1.886040	0.538610	-0.192350
C	-0.815870	1.486770	-0.302310
C	0.474980	1.074940	-0.336710
C	-3.891220	-1.343810	0.019860
C	-4.234850	0.024810	-0.050240
C	-3.235210	0.961830	-0.156100
H	1.524300	-4.099620	-0.059650
H	3.454080	-2.468590	-0.252850
H	2.951940	-0.016580	-0.383930
H	-1.070890	2.551450	-0.357640
H	1.296090	1.796340	-0.420800
H	-4.651900	-2.128720	0.104690
H	-5.287980	0.321030	-0.019780
H	-3.466230	2.031450	-0.212780
N	-2.625560	-1.758920	-0.013080

Waters:

C2 position

O	5.713212	-3.276853	-0.307915
H	6.236135	-3.524863	-1.085304
H	6.307788	-3.428657	0.442235

C3 position

O	4.640428	1.535078	-0.561038
H	5.040354	1.887361	-1.370511
H	5.112040	1.983707	0.157018

C4 position

O	3.093771	3.375723	-0.604901
H	3.502561	3.718104	-1.414195
H	3.574219	3.814597	0.113326

C1-C2 position

O	4.030484	-5.102692	-0.113989
H	4.450037	-5.507350	0.660296
H	4.378384	-5.603556	-0.867243

C2-C3 position

O	5.543415	-0.770747	-0.457978
H	6.160148	-0.605462	0.271139
H	6.088462	-0.701809	-1.256390

C3-C4 position

O	3.728918	2.352300	-0.570047
H	4.202200	2.799163	0.148012
H	4.130543	2.702670	-1.379509

C4-C4 position

O	0.834831	4.452613	-0.566954
H	1.049428	5.065159	0.152786
H	0.977930	4.968650	-1.374741

[Co(phen)(CN)$_2$(H$_2$O)$_2$]

C	1.399915	-2.791781	-0.112727
C	2.458654	-1.882313	-0.261566
C	2.204435	-0.527911	-0.373237
C	0.867871	-0.079209	-0.334356
C	-0.134767	-1.059960	-0.181680
N	0.122767	-2.397114	-0.072746
C	-1.503376	-0.690287	-0.132425
C	-1.884008	0.664239	-0.235665
C	-0.853461	1.647946	-0.390928
C	0.464542	1.291976	-0.438234
C	-3.691968	-1.415926	0.070087
C	-4.156942	-0.094953	-0.025204
C	-3.264201	0.949562	-0.177772
H	1.565426	-3.860609	-0.023064
H	3.473447	-2.264767	-0.286934
H	3.014116	0.186885	-0.489171
H	-1.142814	2.692040	-0.471048
H	1.233763	2.050025	-0.556234
H	-4.366463	-2.257513	0.190912
H	-5.225508	0.085164	0.023602
H	-3.610061	1.976592	-0.252439
N	-2.390200	-1.718135	0.018417
Co	-1.507807	-3.461501	0.122491
C	-3.137594	-4.368680	0.307982
C	-0.548481	-5.069924	0.202991
N	-4.206218	-4.836089	0.425579
N	0.145529	-6.013950	0.240924
O	-1.687410	-3.796455	-2.224201
H	-2.575663	-4.183672	-2.119711
O	-1.397424	-3.386419	2.497863
H	-0.811939	-4.165380	2.512444
H	-1.095938	-4.569631	-2.175858
H	-2.289924	-3.772490	2.564878

Waters:

C2 position

O	-7.491217	0.467072	0.127086
H	-8.029149	0.637033	0.915143
H	-8.122766	0.494238	-0.607556

C3 position

O	-4.342363	4.151159	-0.410535
H	-4.484477	4.783517	0.310032
H	-4.578238	4.641236	-1.212706

C4 position

O	-1.755394	4.902464	-0.640669
H	-1.866882	5.544278	0.076922
H	-1.960100	5.401594	-1.445812

C1-C2 position

O	-6.824806	-1.816343	0.300248
H	-7.427906	-2.087945	-0.408125
H	-7.334289	-1.945149	1.114573

C2-C3 position

O	-6.152878	2.508260	-0.145545
H	-6.651668	2.821906	-0.915041
H	-6.557907	2.964187	0.607696

C3-C4 position

O	-2.984090	4.382489	-0.516728
H	-3.199084	4.878736	-1.320971
H	-3.105866	5.021419	0.201762

C4-C4 position

O	0.631230	4.571391	-0.755354
H	0.736503	5.070539	-1.579404
H	0.829650	5.213055	-0.056650

[Cu(phen)(CN)$_2$(H$_2$O)]

C	1.341157	-3.007499	-0.099476
C	2.419889	-2.109851	-0.191148
C	2.176897	-0.752113	-0.254997
C	0.845578	-0.284061	-0.227369
C	-0.173157	-1.258815	-0.132535
N	0.079315	-2.589394	-0.069897
C	-1.545826	-0.859734	-0.099733
C	-1.886390	0.510253	-0.162903
C	-0.835671	1.479488	-0.258986
C	0.474918	1.098548	-0.289823
C	-3.758037	-1.524618	0.023281
C	-4.190547	-0.187741	-0.035669
C	-3.261029	0.828921	-0.128336
H	1.482068	-4.084188	-0.053064
H	3.431381	-2.500974	-0.210771
H	2.995835	-0.041550	-0.327114
H	-1.103953	2.531055	-0.308182
H	1.262769	1.842889	-0.363674
H	-4.451962	-2.358236	0.094440
H	-5.253793	0.024772	-0.007419
H	-3.574053	1.868447	-0.175312
N	-2.468731	-1.848303	-0.008153
Cu	-1.643744	-3.770883	-0.032634
C	-3.415289	-4.572814	0.103317
C	-0.577269	-5.402809	-0.040462
N	-4.524205	-4.933796	0.017218
N	0.154844	-6.298042	-0.213329
O	-1.838062	-4.016882	-2.493900
H	-1.267858	-4.803497	-2.418182
H	-2.734954	-4.372472	-2.357768

Waters:

C2 position

O	5.576238	-3.330347	-0.252380
H	6.099054	-3.585525	-1.027518
H	6.160521	-3.503263	0.501279

C3 position

O	4.729231	1.462457	-0.479759
H	5.145324	1.809074	-1.283509
H	5.207858	1.892155	0.245200

C4 position

O	2.930757	3.418759	-0.520026
H	3.330098	3.782593	-1.324665
H	3.392374	3.868336	0.203908

C1-C2 position

O	3.820399	-4.967747	-0.093680
H	4.225787	-5.386931	0.680424
H	4.164320	-5.469192	-0.848373

C2-C3 position

O	5.456511	-0.879417	-0.382237
H	6.071792	-0.735845	0.352683
H	6.009257	-0.818925	-1.176026

C3-C4 position

O	3.707585	2.349349	-0.491689
H	4.175018	2.792691	0.232359
H	4.112741	2.706948	-1.296214

C4-C4 position

O	0.713926	4.381324	-0.486577
H	0.909103	4.993894	0.238651
H	0.847806	4.906744	-1.289882

[Pd(phen)$(CN)_2$]

C	1.340349	-2.994206	-0.101446
C	2.420413	-2.107128	-0.239497
C	2.187303	-0.750394	-0.348523
C	0.860384	-0.269734	-0.319605
C	-0.169975	-1.225658	-0.177912
N	0.078570	-2.563824	-0.071624
C	-1.535065	-0.808034	-0.140217
C	-1.860607	0.562700	-0.244237
C	-0.798854	1.513397	-0.387560
C	0.505867	1.114306	-0.423771
C	-3.770192	-1.430383	0.039628
C	-4.174824	-0.089163	-0.057745
C	-3.228762	0.906655	-0.199219
H	1.473571	-4.068110	-0.012106
H	3.428165	-2.507303	-0.258154
H	3.010482	-0.049687	-0.455848
H	-1.054639	2.566009	-0.467493
H	1.302077	1.845066	-0.532782
H	-4.476568	-2.247273	0.152253
H	-5.233442	0.142988	-0.019740
H	-3.523753	1.949411	-0.275832
N	-2.483653	-1.779642	-0.000705
Pd	-1.661360	-3.684811	0.125663
C	-3.387895	-4.556370	0.302326
C	-0.713689	-5.377213	0.226525
N	-4.494395	-4.919751	0.397765
N	0.001683	-6.300210	0.269097

Waters:

C2 position

O	5.472552	-3.319122	-0.296003
H	5.982905	-3.606517	-1.068165
H	6.065405	-3.469808	0.455735

C3 position

O	4.753319	1.433852	-0.683077
H	5.161917	1.748419	-1.503677
H	5.244312	1.885036	0.020236

C4 position

O	2.988021	3.392422	-0.763609
H	3.381285	3.723703	-1.585124
H	3.464983	3.859833	-0.061238

C1-C2 position

O	3.804518	-4.970659	-0.057541
H	4.217562	-5.364542	0.725748
H	4.135063	-5.501251	-0.798152

C2-C3 position

O	5.463570	-0.909507	-0.511399
H	6.089148	-0.745119	0.210348
H	6.006754	-0.881736	-1.313565

C3-C4 position

O	3.749139	2.325123	-0.710070
H	4.230827	2.787354	-0.007496
H	4.147128	2.651224	-1.531382

C4-C4 position

O	0.784105	4.385207	-0.731256
H	0.997306	5.018452	-0.029233
H	0.913378	4.882338	-1.553107
